# A Pneumatic Particle-Blocking Variable-Stiffness Actuator

**DOI:** 10.3390/s23249817

**Published:** 2023-12-14

**Authors:** He Peng, Xia Wang, Dexu Geng, Wenzhi Xu

**Affiliations:** College of Mechanical Engineering, Beihua University, Jilin 132021, China; penghe888@126.com (H.P.); gengdx64@163.com (D.G.); xuwenzhi1212@163.com (W.X.)

**Keywords:** variable stiffness, pneumatic actuation, elastic actuator, flexible robot

## Abstract

In order to improve the stiffness of flexible robots, this paper proposes a variable-stiffness elastic actuator. The actuator integrates the working principles of a pneumatic drive, wedge structure, and particle blockage. The anti-tensile stiffness of the actuator is nonlinearly negatively correlated with the air pressure because of the structural and material properties. The anti-compressive stiffness and lateral stiffness increase nonlinearly as air pressure increases, being 3 and 121 times greater at 0.17 MPa compared to 0 MPa, respectively. Beyond 0.17 MPa, the two stiffnesses of the actuator experience incremental growth due to wedge resistance forces.

## 1. Introduction

Flexible robots have broad application prospects in the field of service robots because of their driving flexibility, unstructured environmental adaptability, and human–computer interaction security [1,2,3]. Researchers have conducted in-depth research on flexible robots, making full use of elastic or soft materials to achieve the flexibility and unstructured environmental adaptability of flexible robots [4,5,6]. However, the traditional flexible robot has low stiffness and weak bearing capacity, which limits the application of flexible robots to a certain extent [7].

In order to improve the stiffness of flexible robots, researchers have proposed variable-stiffness technology. Variable-stiffness structures are being introduced into the design of flexible robots to improve their stiffness and bearing capacity and expand their application range [8,9,10]. At present, the common variable-stiffness technologies include particle blocking, structural interference, and variable-stiffness materials. The particle-blocking variable-stiffness technology developed in the early stages of this research is mostly realized by vacuum negative pressure. The most typical case is the particle-blocking variable-stiffness gripper designed by Amend et al. in 2012. The outer side of the gripper is a film made of soft material, and the inner part is filled with particles. When grasping an object, the particle is in the flow state, so it has a strong tolerance to the clamping object and can adjust its shape to adapt to various objects. After the object has been grabbed, the vacuum of the gripper increases to improve the stiffness and the clamping force [11]. Hua proposed a flexible variable-stiffness manipulator in 2021. It is composed of a strain layer and a limiting layer. The strain layer is a multi-balloon structure made of silicone material. The confined layer is a layer of silica gel with uniform wall thickness and built-in multi-layer materials of the same size. The friction between layered materials is increased through negative-pressure extraction to improve the stiffness of the finger. The bending angle of the finger reaches 86°, and the grasping force of the manipulator reaches 11.89 N [12]. Hauser presented JammJoint in 2017, a compliant and flexible wearable robot, which uses the jamming of granular media to vary its stiffness. It consists of a silicone sleeve with hollow sections that are filled with cubic rubber granules and subjected to different levels of vacuum pressure. The ring stiffness increases more than threefold from 0.0339 to 0.1088 N/mm following a pressure change from 1000 to 200 mbar. Over the same pressure range, the column stiffness changes from 0.0084 to 0.0646 N/mm; the maximum stiffness value is again observed at an intermediate pressure of 400 mbar. The maximum range indicates that an increase in stiffness over a factor of seven is possible [13]. In 2016, Wei proposed the stiffness modulation of ball-joint-based robotic spines using vacuum energy and particles integrated into the ball joint spine to strengthen the spine arm stiffness, and experimental studies have shown that this resulted in a stiffness enhancement of a factor of 13 [14]. Hiroya’s team proposed an electrostatic layer adsorption layered interference variable-stiffness technology in 2017, which increases the adsorption force of the thin layer through the change in the electric field to increase the stiffness [15]. Giannaccini’s team also developed an antagonistic variable-stiffness flexible arm based on the antagonistic principle in 2018. Six contracted artificial muscles are used in parallel, and an elongated pneumatic artificial muscle is placed on the central symmetry axis of three groups of contracted artificial muscles. The pressure gradient of the three groups of contracted artificial muscles is used to realize the spatial bending deformation of the flexible arm, and the pressure of the elongated artificial muscle is applied to realize the stiffness change [16]. Wang developed a three-finger flexible gripper using shape-memory polymer in 2017. The finger has strong fluidity at high temperatures and can adapt to variously shaped objects. After grasping the object, it is treated at low temperatures to improve the stiffness of the gripper. The stiffness of the gripper can be increased by 54 times by adjusting the temperature [17].

In this paper, a variable-stiffness elastic actuator is proposed by combining the working principles of a pneumatic drive, a wedge structure, and particle blocking. It solves the problem of insufficient stiffness and weak bearing capacity of a pneumatic flexible arm and improves its posture retention ability. We established theoretical models for anti-tensile stiffness, anti-compressive stiffness, and lateral stiffness of the actuator and verified them using related experimental tests.

## 2. Working Principle and Design

**Figure 1:**
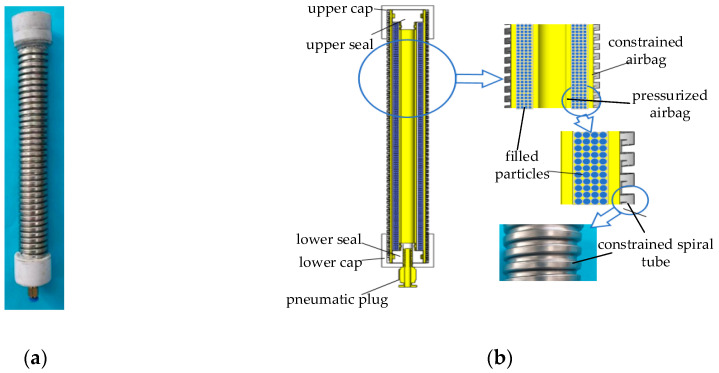
The structure of the variable-stiffness elastic actuator and constrained spiral tube: (**a**) physical object; (**b**) structure.

The variable-stiffness elastic actuator is composed of multiple components arranged coaxially, comprising a pressurized airbag, filling particles, a constrained airbag, and a constrained spiral tube, as illustrated in Figure 1. When subjected to air pressure, the pressurized airbag initially undergoes radial expansion due to its inherent structural and material properties. Axial elongation occurs after the radial deformation reaches its limits. Filling particles are placed between the pressurized and constrained airbag, mainly to facilitate the return to their initial positions after depressurization. At the same time, deformation between the constrained and the pressurized airbags ensures the even distribution of the filling particles during the actuator’s elongation. The constrained spiral tubes are interlocking, allowing for elongation, compression, and bending movements. These tubes fully constrain the internal airbag, preventing any instability. The extent of their elongation determines both the elongation and bending angle of the variable-stiffness elastic actuator. As the spiral tube elongates or bends, the volume of the inner-wall grooves in the nested structure varies in tandem with these changes.

The pressurized airbag and constrained airbag are made of the same hyperelastic silicon–fluorine rubber. The two airbags are installed coaxially, and the middle is filled with diamond sand particles. A stepped sealing plug is situated above the pressurized airbag and anchored to the upper-end cover to ensure an airtight seal. A through-hole in the middle of the lower sealing plug connects to a pneumatic joint, serving as the inlet for pressurized gas. The upper and lower end caps are matched to axially constrain the above parts. Table 1 gives the size and material parameters of the variable-stiffness elastic actuator.

**Figure 2:**
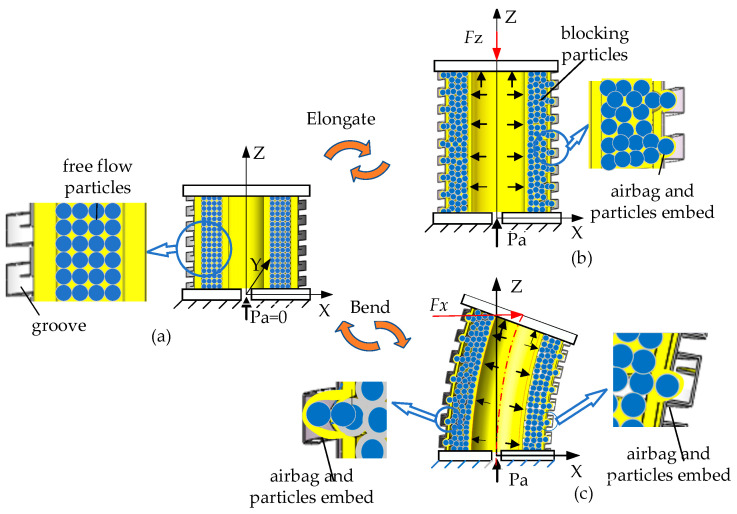
Variable-stiffness principle of the variable-stiffness elastic actuator: (**a**) initial state; (**b**) elongation under axial force; (**c**) elongation under lateral force.

The variable-stiffness principle underlying the actuator is depicted in Figure 2. In its initial state (Figure 2a), the pressurized airbag is unpressurized, allowing the filling particles to flow dynamically, and the groove in the spiral tube is half open. When the variable-stiffness elastic actuator is pressurized, the filling particles are squeezed into a blocked state, and the groove enlarges as the actuator elongates axially. The filling particles and constrained airbag become embedded in this expanded groove to form a wedge structure. At this time, the axial force is applied to the actuator, as illustrated in Figure 2b; when the axial force is pressed down to the actuator, the reason for the change in the stiffness includes the blocked particle and wedge resistance force. When the axial force is pulled up the actuator, there is no wedge resistance force, so the variable-stiffness principle is only based on particle blockage. When the lateral force is applied to the actuator, as shown in Figure 2c, the wedge structure on the tension side of the constraint spiral tube does not produce resistance force, and the wedge resistance force exists on the compression side. The reason for the change in the stiffness includes the blocked particle and the wedge resistance force on the compression side of the spiral tube.

## 3. Theoretical Modeling

The variable-stiffness elastic actuator is applied to the flexible arm as a driving device, and the variable-stiffness function of the flexible arm is realized at the same time. During the movement of the flexible arm, the actuator mainly bears axial force and lateral force. Therefore, the anti-tensile, anti-compressive, and lateral stiffnesses are primarily studied.

### 3.1. Anti-Tensile Stiffness at Any Position of the Variable-Stiffness Elastic Actuator

**Figure 3:**
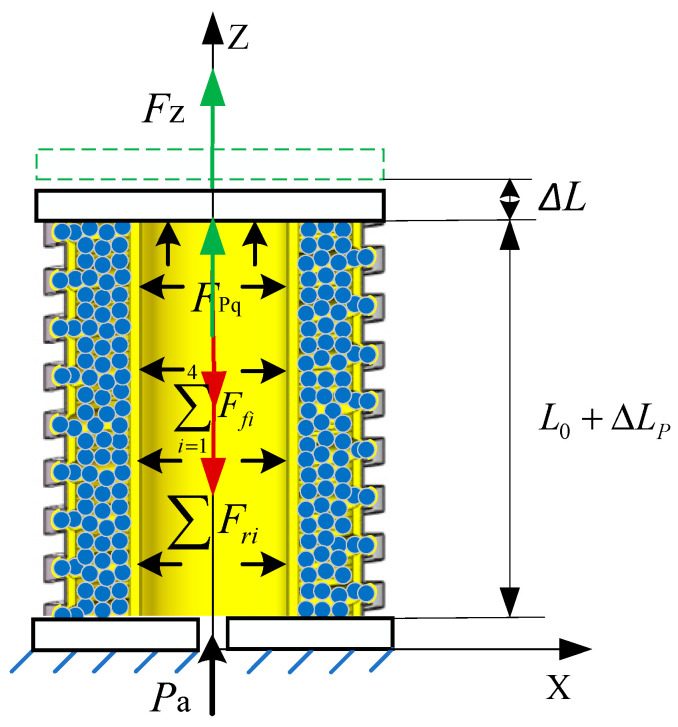
Force analysis of the variable-stiffness elastic actuator under action of *F*_z_.

As illustrated in Figure 3, the actuator undergoes axial elongation, $\Delta L_{P}$, due to applied air pressure. In this state, the axial force $F_{z}$, is applied at the center of the upper-end cover, causing the actuator to elongate and deform. As a result, the center point of the upper-end cover moves ΔL along the axis direction. The friction includes frictional force in various areas: between the constrained airbag and the spiral tube, among the filling particles and the constrained airbag, and finally, between the filling particles and the pressurized airbag. The constrained airbag, pressurized airbag, and spiral tube generate deformation resistance force.

The static equilibrium equation of the variable-stiffness elastic actuator is as follows:

(1) $$F_{\mathrm{Pq}}+F_{z}=\sum_{i=1}^{4}F_{fi}+\sum F_{ri}$$

In the formula, $F_{\mathrm{Pq}}$ is the driving force; $\sum_{i=1}^{4}F_{fi}$ is the friction resistance forces between the constrained airbag and the constrained spiral tube, between the filling particles, between the filling particles and the constrained airbag, and between the filling particles and the pressurized airbag; and $\sum F_{ri}$ is the axial deformation resistance force of the constrained airbag, pressurized airbag, and constrained spiral tube.

1. Driving force

The driving force is given by:

(2) $$F_{Pq}=P_{a}{S^{\prime }}_{c}$$

where $P_{a}$ represents the air pressure applied to the pressurized airbag, and ${S^{\prime }}_{c}$ denotes the cross-sectional area of the cavity after the pressurized airbag has deformed.

Both the constrained and pressurized airbags are fabricated from silicon–fluorine rubber—a highly elastic and incompressible material—ensuring that the annular volume remains constant after deformation. The constrained spiral tube is made of a rigid material, so its internal cross-sectional area remains unchanged before and after deformation. Consequently, any volume change in the inner cavity of the constrained spiral tube directly reflects the volume change in the inner cavity of the pressurized airbag.

(3) $$S_{L}\left(\Delta L_{P}+\Delta L\right)={S^{\prime }}_{c}(L_{0}+\Delta L_{P}+\Delta L)-S_{c}L_{0}$$

where $S_{L}$ represents the cross-sectional area of the inner wall of the spiral tube, $L_{0}$ denotes the initial effective length of the variable-stiffness elastic actuator, and $S_{c}$ symbolizes the initial cross-sectional area of the cavity in the pressurized airbag.

(4) $$S_{L}=\frac{\pi D_{L2}^{2}}{4}$$

where $D_{L2}$ stands for the diameter of the inner wall of the spiral tube.

(5) $$S_{c}=\frac{\pi D_{c2}^{2}}{4}$$

where $D_{c2}$ represents the initial diameter of the inner wall of the pressurized airbag.

The effective working area of the pressurized airbag is defined by the cross-sectional area of its cavity.

(6) $${S^{\prime }}_{c}=\frac{S_{L}\left(\Delta L_{P}+\Delta L\right)+S_{c}L_{0}}{L_{0}+\Delta L_{P}+\Delta L}=\frac{\pi D_{L2}^{2}\left(\Delta L_{P}+\Delta L\right)+\pi D_{c2}^{2}L_{0}}{4(L_{0}+\Delta L_{P}+\Delta L)}$$

After the inflation of the pressurized airbag, the axial extension of the actuator causes the spiral tube groove to become larger, and the particles and constrained airbags are embedded in the groove, resulting in an increase in the cross-sectional area of the pressurized airbag. Therefore, it is necessary to introduce a cross-sectional area correction coefficient $K_{A}$ on the basis of the aforementioned pressure axial force model. The correction coefficient is related to factors such as particle motion trajectory and groove volume change, and can be obtained through experimental analysis. Substituting Equation (6) into Equation (2), the driving force is

(7) $$F_{Pq}=\frac{K_{A}P_{a}\pi \left[D_{L2}^{2}\left(\Delta L_{P}+\Delta L\right)+D_{c2}^{2}L_{0}\right]}{4(L_{0}+\Delta L_{P}+\Delta L)}$$

2. Friction resistance force

**Figure 4:**
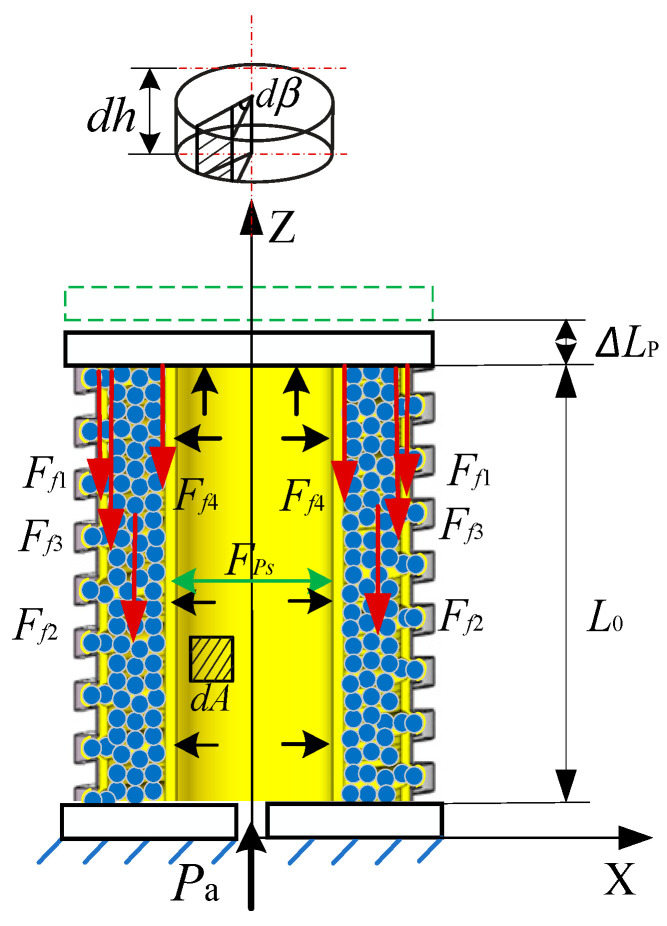
The friction analysis of the variable-stiffness elastic actuator.

As the actuator expands under pressure, a positive pressure is generated on the inner-wall surface of the pressurized airbag, generating friction resistance force among the actuator’s components (Figure 4).

(8) $$dF_{f}=fdF_{Ps}=fP_{a}dA=fP_{a}\sqrt{\frac{\left(D_{L2}^{2}\Delta L_{P}+D_{c2}{}^{2}L_{0}\right)}{4(L_{0}+\Delta L_{P})}}d\beta dh$$

where $dF_{Ps}$ is the infinitesimal positive pressure element of the pressurized airbag, and dA represents the infinitesimal area element after the airbag is pressurized and deformed. dβ represents the infinitesimal angle element on the inner-wall surface. dh denotes the infinitesimal height element on the inner-wall surface. f is the friction coefficient.

The friction force between the constrained airbag and the spiral tube is

(9) $$\begin{array}{l} F_{f1}=f_{1}P_{a}\sqrt{\frac{D_{L2}{}^{2}\Delta L_{P}+D_{c2}{}^{2}L_{0}}{4(L_{0}+\Delta L_{P})}}\int_{0}^{L_{0}+\Delta L_{P}+\Delta L}\int_{0}^{2\pi }d\beta dh\\ =f_{1}P_{a}\pi (L_{0}+\Delta L_{P}+\Delta L)\sqrt{\frac{D_{L2}{}^{2}\Delta L_{P}+D_{c2}{}^{2}L_{0}}{L_{0}+\Delta L_{P}}} \end{array}$$

where $f_{1}$ is the friction coefficient between the constrained airbag and spiral tube, and the axial friction force among the filling particles is

(10) $$\begin{array}{l} F_{f2}=nf_{2}P_{a}\sqrt{\frac{D_{L2}{}^{2}\Delta L_{P}+D_{c2}{}^{2}L_{0}}{4(L_{0}+\Delta L_{P})}}\int_{0}^{L_{0}+\Delta L_{P}+\Delta L}\int_{0}^{2\pi }d\beta dh\\ =nf_{2}P_{a}\pi (L_{0}+\Delta L_{P}+\Delta L)\sqrt{\frac{D_{L2}{}^{2}\Delta L_{P}+D_{c2}{}^{2}L_{0}}{L_{0}+\Delta L_{P}}} \end{array}$$

where $f_{2}$ signifies the friction coefficient between the particles, and n denotes the particle correction coefficient.

The friction force among the constrained airbag and particles is

(11) $$\begin{array}{l} F_{f3}=nf_{3}P_{a}\sqrt{\frac{D_{L2}{}^{2}\Delta L_{P}+D_{c2}{}^{2}L_{0}}{4(L_{0}+\Delta L_{P})}}\int_{0}^{L_{0}+\Delta L_{P}+\Delta L}\int_{0}^{2\pi }d\beta dh\\ =nf_{3}P_{a}\pi (L_{0}+\Delta L_{P}+\Delta L)\sqrt{\frac{D_{L2}{}^{2}\Delta L_{P}+D_{c2}{}^{2}L_{0}}{L_{0}+\Delta L_{P}}} \end{array}$$

where $f_{3}$ is the friction coefficient between the particles and the constrained airbag.

The friction between the particles and the pressurized airbag is

(12) $$\begin{array}{l} F_{f4}=nf_{4}P_{a}\sqrt{\frac{D_{L2}{}^{2}\Delta L_{P}+D_{c2}{}^{2}L_{0}}{4(L_{0}+\Delta L_{P})}}\int_{0}^{L_{0}+\Delta L_{P}+\Delta L}\int_{0}^{2\pi }d\beta dh\\ =nf_{4}P_{a}\pi (L_{0}+\Delta L_{P}+\Delta L)\sqrt{\frac{D_{L2}{}^{2}\Delta L_{P}+D_{c2}{}^{2}L_{0}}{L_{0}+\Delta L_{P}}} \end{array}$$

where $f_{4}$ is the friction coefficient between the particles and the pressurized airbag.

3. Deformation resistance force

The variable-stiffness elastic actuator moves ΔL axial elongation under the action of external force $F_{z}$. According to the principle of elastic deformation, the axial deformation resistance force of the constrained airbag is [18]

(13) $$F_{\mathrm{ry}}=\frac{\pi E(D_{y1}{}^{2}-D_{y2}{}^{2})L_{0}\left(\Delta L_{P}+\Delta L\right)}{4{\left(L_{0}+\Delta L_{P}+\Delta L\right)}^{2}}$$

where E denotes the elastic modulus of the airbag, $D_{y1}$ is the initial diameter of the outer wall of the constrained airbag, and $D_{y2}$ is the initial diameter of the inner wall of the constrained airbag.

The axial deformation resistance force of the pressurized airbag is

(14) $$F_{\mathrm{rc}}=\frac{E\pi (D_{c1}{}^{2}-D_{c2}{}^{2})L_{0}\left(\Delta L_{P}+\Delta L\right)}{4{\left(L_{0}+\Delta L_{P}+\Delta L\right)}^{2}}$$

where $D_{c1}$ denotes the initial outer diameter of the pressurized airbag, and $D_{c2}$ is the initial diameter of the inner wall of the pressurized airbag.

The resistance force generated by the constrained spiral tube is smaller than the above force and can be ignored. Therefore, substituting Equations (7) and (9)–(14) into Equation (1), the relationship between external load *F*_z_ and the axial elongation is

(15) $$\begin{array}{l} f(F_{z},\Delta L)=0\\ =F_{z}+\frac{K_{A}P_{a}\pi \left[D_{L2}{}^{2}\left(\Delta L_{P}+\Delta L\right)+D_{c2}{}^{2}L_{0}\right]}{4(L_{0}+\Delta L_{P}+\Delta L)}-\frac{\pi E\left[\left(D_{y1}{}^{2}-D_{y2}{}^{2})+(D_{c1}{}^{2}-D_{c2}{}^{2}\right)\right]L_{0}\left(\Delta L_{P}+\Delta L\right)}{4{\left(L_{0}+\Delta L_{P}+\Delta L\right)}^{2}}\\ -(f_{1}+nf_{2}+nf_{3}+nf_{4})\pi P_{a}(L_{0}+\Delta L_{P}+\Delta L)\sqrt{\frac{D_{L2}{}^{2}\Delta L_{P}+D_{c2}{}^{2}L_{0}}{L_{0}+\Delta L_{P}}} \end{array}$$

The anti-tensile stiffness of the variable-stiffness elastic actuator is

(16) $$K_{L}(P_{a},\Delta L,\Delta L_{P})=\frac{F_{z}(P_{a},\Delta L,\Delta L_{P})}{\Delta L}$$

When $0\leq \Delta L_{P}<0.2L$, the anti-tensile stiffness is calculated according to Equation (16). When $\Delta L_{P}=0.2L$, the variable-stiffness elastic actuator reaches maximum elongation, and the anti-tensile stiffness depends on the anti-tensile strength of the constrained spiral tube.

### 3.2. Anti-Compressive Stiffness at Any Position of the Variable-Stiffness Elastic Actuator

**Figure 5:**
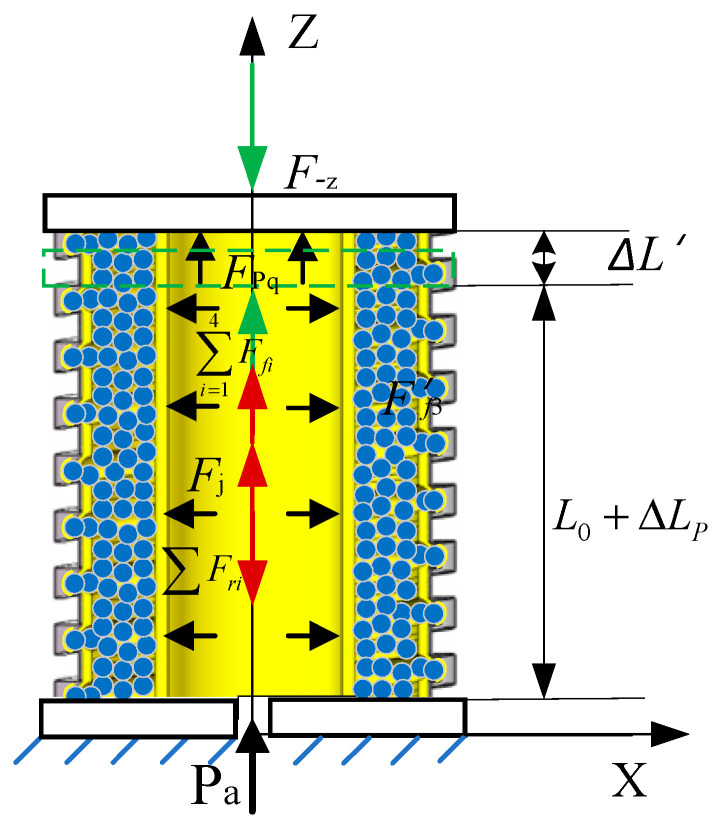
Force analysis of the variable-stiffness elastic actuator under action of *F_-_*_z_.

As illustrated in Figure 5, the actuator undergoes axial elongation, $\Delta L_{P}$, due to applied air pressure. In this state, the axial force *F*_-z_ is applied at the center of the upper-end cover, causing the actuator to elongate and deform. As a result, the center point of the upper-end cover moves $\Delta L^{\prime }$ along the axis direction. At this time, the internal friction contributing to the resistance moment mainly includes frictional interactions in various regions: between the constrained airbag and the spiral tube, among the filling particles and the constrained airbag, and finally, between the filling particles and the pressurized airbag. The constrained airbag, pressurized airbag, and spiral tube generate deformation resistance force. The wedge resistance force $F_{j}$ is generated by filling particles and constraining the airbag in the spiral groove.

According to the static equilibrium equation,

(17) $$F_{-z}+\sum F_{ri}=F_{\mathrm{Pq}}+\sum_{i=1}^{4}F_{fi}+F_{j}$$

1. Driving force

The variable-stiffness elastic actuator is compressed under the action of external force, and the air pressure value remains unchanged. Therefore, when the elongation changes to $\Delta L_{P}-\Delta L$, the cross-sectional area of the inner cavity changes. According to Equation (7),

(18) $$F_{Pq}=\frac{K_{A}P_{a}\pi \left[D_{L2}^{2}\left(\Delta L_{P}-\Delta L^{\prime }\right)+D_{c2}^{2}L_{0}\right]}{4(L_{0}+\Delta L_{P}-\Delta L^{\prime })}$$

2. Friction resistance force

The friction force between the constrained airbag and the spiral tube is constrained to

(19) $$\begin{array}{l} F_{f1}=f_{1}P_{a}\sqrt{\frac{\left(D_{L2}{}^{2}\Delta L_{P}+D_{c2}{}^{2}L_{0}\right)}{4(L_{0}+\Delta L_{P})}}\int_{0}^{L_{0}+\Delta L_{P}-\Delta L^{\prime }}\int_{0}^{2\pi }d\beta dh\\ =f_{1}P_{a}\pi \left(L_{0}+\Delta L_{P}-\Delta L^{\prime }\right)\sqrt{\frac{\left(D_{L2}{}^{2}\Delta L_{P}+D_{c2}{}^{2}L_{0}\right)}{\left(L_{0}+\Delta L_{P}\right)}} \end{array}$$

The axial friction force among the filling particles is

(20) $$\begin{array}{l} F_{f2}=nf_{2}P_{a}\sqrt{\frac{\left(D_{L2}{}^{2}\Delta L_{P}+D_{c2}{}^{2}L_{0}\right)}{4(L_{0}+\Delta L_{P})}}\int_{0}^{L_{0}+\Delta L_{P}-\Delta L^{\prime }}\int_{0}^{2\pi }d\beta dh\\ =nf_{2}P_{a}\pi \left(L_{0}+\Delta L_{P}-\Delta L^{\prime }\right)\sqrt{\frac{\left(D_{L2}{}^{2}\Delta L_{P}+D_{c2}{}^{2}L_{0}\right)}{\left(L_{0}+\Delta L_{P}\right)}} \end{array}$$

The friction force among the particles and the constrained airbag is equal to

(21) $$\begin{array}{l} F_{f3}=nf_{3}P_{a}\sqrt{\frac{\left(D_{L2}{}^{2}\Delta L_{P}+D_{c2}{}^{2}L_{0}\right)}{4(L_{0}+\Delta L_{P})}}\int_{0}^{L_{0}+\Delta L_{P}-\Delta L^{\prime }}\int_{0}^{2\pi }d\beta dh\\ =nf_{3}P_{a}\pi \left(L_{0}+\Delta L_{P}-\Delta L^{\prime }\right)\sqrt{\frac{\left(D_{L2}{}^{2}\Delta L_{P}+D_{c2}{}^{2}L_{0}\right)}{\left(L_{0}+\Delta L_{P}\right)}} \end{array}$$

The friction between the particles and the pressurized airbag is

(22) $$\begin{array}{l} F_{f4}=nf_{4}P_{a}\sqrt{\frac{\left(D_{L2}{}^{2}\Delta L_{P}+D_{c2}{}^{2}L_{0}\right)}{4(L_{0}+\Delta L_{P})}}\int_{0}^{L_{0}+\Delta L_{P}-\Delta L^{\prime }}\int_{0}^{2\pi }d\beta dh\\ =nf_{4}P_{a}\pi \left(L_{0}+\Delta L_{P}-\Delta L^{\prime }\right)\sqrt{\frac{\left(D_{L2}{}^{2}\Delta L_{P}+D_{c2}{}^{2}L_{0}\right)}{\left(L_{0}+\Delta L_{P}\right)}} \end{array}$$

3. Deformation resistance force

According to Equation (13), the deformation resistance of the constrained airbag is

(23) $$F_{\mathrm{ry}}=\frac{\pi E(D_{y1}{}^{2}-D_{y2}{}^{2})L_{0}\left(\Delta L_{P}-\Delta L^{\prime }\right)}{4{\left(L_{0}+\Delta L_{P}-\Delta L^{\prime }\right)}^{2}}$$

According to the Equation (14), the deformation resistance of the pressurized airbag is

(24) $$F_{\mathrm{rc}}=\frac{E\pi (D_{c1}{}^{2}-D_{c2}{}^{2})L_{0}\left(\Delta L_{P}-\Delta L^{\prime }\right)}{4{\left(L_{0}+\Delta L_{P}-\Delta L^{\prime }\right)}^{2}}$$

4. Wedge resistance moment

**Figure 6:**
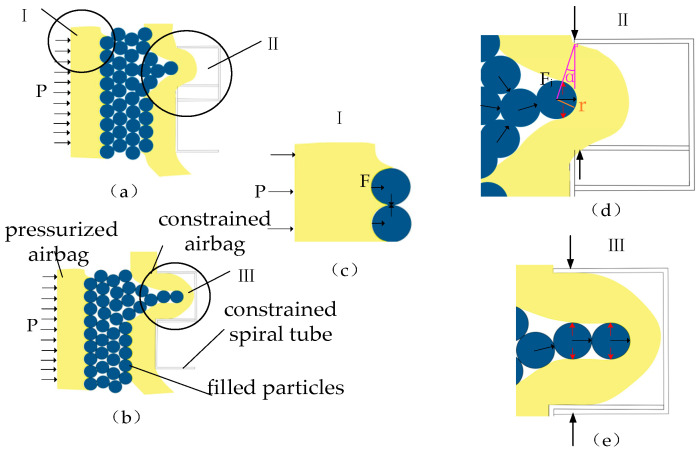
Mechanism analysis of wedge resistance force: (**a**) embedded volume of particles is less than half; (**b**) particles are completely embedded; (**c**) force analysis of unit-filled particles; (**d**) resistance force analysis of wedge mechanism; and (**e**) force analysis of completely embedded particles.

The magnitude of the wedge resistance force is influenced by the volume of the filler particles and the depth to which the constrained airbag is embedded in the groove. When the embedded depth of the filled particles is less than the radius of the particles (Figure 6a), the force model can be analyzed using wedge-clamping mechanisms, as depicted in Figure 6d. When the filling particles are completely embedded in the groove (Figure 6b), the force model is as shown in Figure 6e. Essentially, the force magnitude depends on the anti-compressive strength of the embedded particles and the constrained airbag within the groove.

The force exerted by the pressurized airbag on unit-filling particles when their embedding depth is less than the particle radius is presented in Figure 6c and defined as:

(25) $$F=P_{a}\pi r^{2}$$

where *F* denotes the pressure applied to a unit of particles by the pressurized airbag, and r represents the radius of the unit-filled particle.

For simplicity, we assume a uniform interaction force among the filling particles and designated this uniform force as *F*. These particles, along with the constrained airbag, are embedded in the groove of the spiral tube, functioning as a wedge-clamping mechanism (Figure 6d). The corresponding wedge resistance force is

(26) $$F_{j}=\frac{F}{2\tan(\alpha +\phi )}$$

where φ denotes the friction angle, and α represents the wedge lift angle, associated with the inlet pressure or particle embedding depth.

Therefore,

(27) $$F_{j}=\frac{P_{a}\pi r^{2}}{2\tan(\alpha +\phi )}$$

Thus, substituting Equations (18)–(24) and (27) into Equation (17), the relationship between the elongation and the external force is

(28) $$\begin{array}{l} f(F_{-z},\Delta L^{\prime })=0\\ ={F^{\prime }}_{z}-\frac{K_{A}\pi P_{a}\left[D_{L2}{}^{2}\left(\Delta L_{P}-\Delta L^{\prime }\right)+D_{c2}{}^{2}L_{0}\right]}{4(L_{0}+\Delta L_{P}-\Delta L^{\prime })}-\frac{N_{k}P_{a}\pi r^{2}}{2\tan(\alpha +\phi )}\\ -\frac{E\pi \left[\left(D_{y1}{}^{2}-D_{y2}{}^{2})+(D_{c1}{}^{2}-D_{c2}{}^{2}\right)\right]L_{0}\left(\Delta L_{P}-\Delta L^{\prime }\right)}{4\left(L_{0}+\Delta L_{P}-\Delta L^{\prime }\right)}\\ (f_{1}+nf_{2}+nf_{3}+nf_{4})\pi P_{a}\pi \left(L_{0}+\Delta L_{P}-\Delta L^{\prime }\right)\sqrt{\frac{\left(D_{L2}{}^{2}\Delta L_{P}+D_{c2}{}^{2}L_{0}\right)}{\left(L_{0}+\Delta L_{P}\right)}} \end{array}$$

where *N*_k_ is the total number of particles embedded in the groove.

When the embedded groove depth of the filling particles is less than the particle radius, the anti-compressive stiffness of the variable-stiffness elastic actuator is

(29) $$K_{Y}(P_{a},\Delta L^{\prime },\Delta L_{P})=\frac{F_{-z}(P_{a},\Delta L^{\prime },\Delta L_{P})}{\Delta L^{\prime }}$$

When $0\leq \Delta L_{P}<0.1L$, the anti-compressive stiffness of the variable-stiffness elastic actuator is calculated according to Equation (29). When $\Delta L_{P}\geq 0.1L$, the filling particles are completely embedded in the groove of the spiral tube, and the anti-compressive stiffness depends on the anti-compressive strength of the filling particles and the constrained airbag in the embedded groove.

### 3.3. Lateral Stiffness at Any Position of the Variable-Stiffness Elastic Actuator

**Figure 7:**
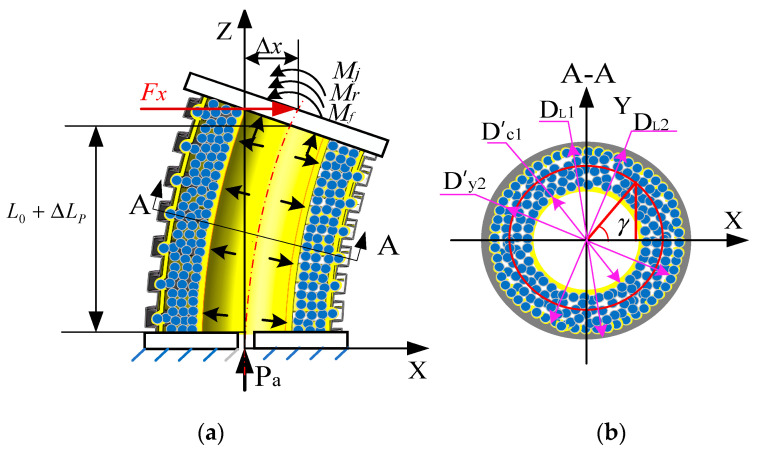
Bending force model of the variable-stiffness elastic actuator: (**a**) forced model; (**b**) force arm model.

The lateral stiffness of a flexible robot is generally considered to be weak. Therefore, when integrating a variable-stiffness actuator into such a robot, the focus predominantly shifts to studying the lateral stiffness. Figure 7a illustrates the bending model of the variable-stiffness elastic actuator, which undergoes axial elongation due to applied air pressure. In this state, a lateral force, *F*_x_, is applied at the center of the upper-end cover, causing the actuator to bend and deform. As a result, the center point of the upper-end cover moves Δx in the direction of *F*_x_. When subjected to this external load *F*_x_, the variable-stiffness elastic actuator behaves akin to a cantilever beam: it elongates on its left side and compresses on the right. This deformation produces a friction resistance moment at the upper-end cover of the actuator. The various components—constrained airbags, pressurized airbags, and spiral tubes—contribute to deformation resistance moments. Additionally, a wedge resistance moment forms on the compressed side of the spiral tube. It is worth noting that while the driving force has an effect on the local lateral stiffness of the actuator, it does not significantly influence the overall lateral stiffness [19].

According to the moment balance equation of the upper cover:

(30) $$M_{x}=\sum_{i=1}^{4}M_{fi}+\sum_{i=1}^{3}M_{ri}+M_{j}$$

where $M_{x}$ is the resistance moment due to the external force *F*_x_, $M_{fi}$ is friction-induced resistance moment, $M_{ri}$ is the deformation resistance moment, and $M_{j}$ is the wedge resistance moment.

1. External moment

The driving moment generated by the external load *F*_x_ is

(31) $$M_{x}=F_{x}\left(L_{0}+\Delta L_{P}\right)$$

2. Friction resistance moment

**Figure 8:**
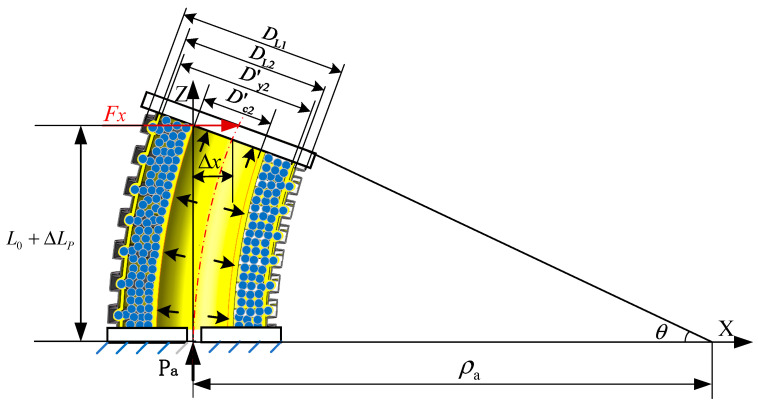
Bending geometric relationship of the variable-stiffness elastic actuator.

Due to the bending angle, θ is small under the action of the lateral force *F*_x_. Considering the bending geometry of the actuator shown in Figure 8, the end-face rotation angle is

(32) $$\theta =\frac{\Delta x}{L_{0}+\Delta L_{p}}$$

The bending radius of curvature is

(33) $$\rho_{a}=\frac{L_{0}+\Delta L_{p}}{\theta }=\frac{{\left(L_{0}+\Delta L_{p}\right)}^{2}}{\Delta x}$$

When the actuator is subjected to the external force *F*_x_, the left side of the pressurized airbag elongates, while the right side compresses. The inner-wall diameter remains unchanged. The elongated length on the left side of the inner wall of the pressurized airbag is

(34) $$L_{\mathrm{cz}}=\left(\rho_{a}+\frac{D_{c2}}{2}\right)\theta =L_{0}+\Delta L_{P}+\frac{D_{c2}\Delta x}{2\left(L_{0}+\Delta L_{P}\right)}$$

The length on the right side of the pressurized airbag is

(35) $$L_{\mathrm{cy}}=\left(\rho_{a}-\frac{D_{c2}}{2}\right)\theta =L_{0}+\Delta L_{P}-\frac{D_{c2}\Delta x}{2\left(L_{0}+\Delta L_{P}\right)}$$

The force arms of the friction resistance moment are different due to the different diameters of the constrained airbag, pressurized airbag, and constrained spiral tube. And each friction force is different in the circumferential range, as shown in Figure 7b.

Therefore,

(36) $$\begin{array}{l} M_{f1}=nf_{1}P_{a}\sqrt{\frac{D_{L2}{}^{2}\Delta L_{P}+D_{c2}{}^{2}L_{0}}{4(L_{0}+\Delta L_{P})}}\\ \left(\int_{-\frac{\pi }{2}}^{\frac{\pi }{2}}\int_{0}^{L_{0}+\Delta L_{P}+\frac{D_{c2}}{2}\theta }\int_{-\frac{\pi }{2}}^{\frac{\pi }{2}}\frac{D_{L2}}{2}\cos\gamma d\beta dhd\gamma +\int_{-\frac{\pi }{2}}^{\frac{\pi }{2}}\int_{0}^{L_{0}+\Delta L_{P}-\frac{D_{c2}}{2}\theta }\int_{-\frac{\pi }{2}}^{\frac{\pi }{2}}\frac{D_{L2}}{2}\cos\gamma d\beta dhd\gamma \right)\\ =f_{1}P_{a}\pi D_{L2}\sqrt{\left(D_{L2}{}^{2}\Delta L_{P}+D_{c2}{}^{2}L_{0}\right)(L_{0}+\Delta L_{P})} \end{array}$$

(37) $$\begin{array}{l} M_{f2}=nf_{2}P_{a}\sqrt{\frac{D_{L2}{}^{2}\Delta L_{P}+D_{c2}{}^{2}L_{0}}{4(L_{0}+\Delta L_{P})}}\\ \left(\int_{-\frac{\pi }{2}}^{\frac{\pi }{2}}\int_{0}^{L_{0}+\Delta L_{P}+\frac{D_{c2}}{2}\theta }\int_{-\frac{\pi }{2}}^{\frac{\pi }{2}}\frac{{D^{\prime }}_{y2}+{D^{\prime }}_{c1}}{2}\cos\gamma d\beta dhd\gamma +\int_{-\frac{\pi }{2}}^{\frac{\pi }{2}}\int_{0}^{L_{0}+\Delta L_{P}-\frac{D_{c2}}{2}\theta }\int_{-\frac{\pi }{2}}^{\frac{\pi }{2}}\frac{{D^{\prime }}_{y2}+{D^{\prime }}_{c1}}{2}\cos\gamma d\beta dhd\gamma \right)\\ =nf_{2}P_{a}\pi \left({D^{\prime }}_{y2}+{D^{\prime }}_{c1}\right)\sqrt{\left(D_{L2}{}^{2}\Delta L_{P}+D_{c2}{}^{2}L_{0}\right)(L_{0}+\Delta L_{P})} \end{array}$$

where ${D^{\prime }}_{c1}$ is the outer-wall diameter of the pressurized airbag after deformation, ${D^{\prime }}_{c1}=\sqrt{\frac{D_{c1}{}^{2}L_{0}+D_{L2}{}^{2}\Delta L_{P}}{\left(L_{0}+\Delta L_{P}\right)}}$, and ${D^{\prime }}_{y2}$ is the inner-wall diameter of the constrained airbag after deformation, ${D^{\prime }}_{y2}=\sqrt{\frac{D_{y1}{}^{2}\Delta L_{P}+D_{y2}{}^{2}L_{0}}{\left(L_{0}+\Delta L_{P}\right)}}$.

(38) $$\begin{array}{l} M_{f3}=nf_{3}P_{a}\sqrt{\frac{D_{L2}{}^{2}\Delta L_{P}+D_{c2}{}^{2}L_{0}}{4(L_{0}+\Delta L_{P})}}\\ \left(\int_{-\frac{\pi }{2}}^{\frac{\pi }{2}}\int_{0}^{L_{0}+\Delta L_{P}+\frac{D_{c2}}{2}\theta }\int_{-\frac{\pi }{2}}^{\frac{\pi }{2}}\frac{{D^{\prime }}_{y2}}{2}\cos\gamma d\beta dhd\gamma +\int_{-\frac{\pi }{2}}^{\frac{\pi }{2}}\int_{0}^{L_{0}+\Delta L_{P}-\frac{D_{c2}}{2}\theta }\int_{-\frac{\pi }{2}}^{\frac{\pi }{2}}\frac{{D^{\prime }}_{y2}}{2}\cos\gamma d\beta dhd\gamma \right)\\ =nf_{3}P_{a}\pi {D^{\prime }}_{y2}\sqrt{\left(D_{L2}{}^{2}\Delta L_{P}+D_{c2}{}^{2}L_{0}\right)(L_{0}+\Delta L_{P})} \end{array}$$

(39) $$\begin{array}{l} M_{f4}=nf_{4}P_{a}\sqrt{\frac{D_{L2}{}^{2}\Delta L_{P}+D_{c2}{}^{2}L_{0}}{4(L_{0}+\Delta L_{P})}}\\ \left(\int_{-\frac{\pi }{2}}^{\frac{\pi }{2}}\int_{0}^{L_{0}+\Delta L_{P}+\frac{D_{c2}}{2}\theta }\int_{-\frac{\pi }{2}}^{\frac{\pi }{2}}\frac{{D^{\prime }}_{c1}}{2}\cos\gamma d\beta dhd\gamma +\int_{-\frac{\pi }{2}}^{\frac{\pi }{2}}\int_{0}^{L_{0}+\Delta L_{P}-\frac{D_{c2}}{2}\theta }\int_{-\frac{\pi }{2}}^{\frac{\pi }{2}}nf_{4}P_{a}\frac{{D^{\prime }}_{c1}}{2}\cos\gamma d\beta dhd\gamma \right)\\ =nf_{4}P_{a}\pi {D^{\prime }}_{c1}\sqrt{\left(D_{L2}{}^{2}\Delta L_{P}+D_{c2}{}^{2}L_{0}\right)(L_{0}+\Delta L_{P})} \end{array}$$

3. Deformation resistance moment

The deformation of the annular section of the constrained airbag and pressurized airbag is stable because of the radial constraint of the constrained spiral tube. Under the action of external force, the actuator behaves according to the plane-bending model of an elastic beam. According to the Euler–Bernoulli beam theory, the axial deformation resistance moment generated by the constrained airbag is [18,20]

(40) $$M_{\mathrm{ry}}=\frac{E\pi (D_{y1}^{2}-D_{y2}^{2})\left[(D_{y1}^{2}+D_{y2}^{2})L_{0}{}^{2}+2D_{y1}^{2}L_{0}\Delta L_{P}\right]\Delta x}{64{\left(L_{0}+\Delta L_{P}\right)}^{4}}$$

The axial deformation resistance moment generated by the pressurized airbag under the action of external force is

(41) $$M_{\mathrm{rc}}=\frac{E\pi (D_{c1}^{2}-D_{c2}^{2})\left[(D_{c1}^{2}+D_{c2}^{2})L_{0}{}^{2}+2D_{L2}^{2}L_{0}\Delta L_{P}\right]\Delta x}{64{\left(L_{0}+\Delta L_{P}\right)}^{4}}$$

Therefore, substituting Equations (31) and (36)–(41) into Equation (30), the relationship between the bending angle and the external force is

(42) $$\begin{array}{l} f\left(F_{x},\theta \right)=0\\ =F_{x}(L_{0}+\Delta L_{P})-\frac{P_{a}r^{2}\left(D_{L1}+D_{L2}\right)}{2\tan(\alpha +\phi )}\\ -\frac{E\pi \left(D_{y1}^{2}-D_{y2}^{2}\right)\left(D_{c1}^{2}-D_{c2}^{2}\right)\left\{\left[\left(D_{y1}^{2}+D_{y2}^{2}\right)+\left(D_{c1}^{2}+D_{c2}^{2}\right)\right]L_{0}^{2}+2D^{2}{}_{y1}L_{0}\Delta L_{P}\right\}\Delta x}{64{\left(L_{0}+\Delta L_{P}\right)}^{4}}\\ \left[fD_{L2}+nf_{2}\left(\sqrt{\frac{D_{c1}{}^{2}L_{0}+D_{L2}{}^{2}\Delta L_{P}}{L_{0}+\Delta L_{P}}}+\sqrt{\frac{D_{y1}{}^{2}\Delta L_{P}+D_{y2}{}^{2}L_{0}}{L_{0}+\Delta L_{P}}}\right)+nf_{3}\sqrt{\frac{D_{y1}{}^{2}\Delta L_{P}+D_{y2}{}^{2}L_{0}}{L_{0}+\Delta L_{P}}}+nf_{4}\sqrt{\frac{D_{c1}{}^{2}L_{0}+D_{L2}{}^{2}\Delta L_{P}}{L_{0}+\Delta L_{P}}}\right]\\ P_{a}\pi \sqrt{\left(D_{L2}{}^{2}\Delta L_{P}+D_{c2}{}^{2}L_{0}\right)\left(L_{0}+\Delta L_{P}\right)} \end{array}$$

The lateral stiffness of the variable-stiffness elastic actuator is

(43) $$K_{x}(P_{a},\Delta x,\Delta L_{P})=\frac{F_{x}(P_{a},\Delta x,\Delta L_{P})}{\Delta x}$$

When $0\leq \Delta L_{P}<0.1L$, the lateral stiffness is calculated according to Equation (43). When $\Delta L_{P}\geq 0.1L$, the resistance moment of the wedge depends on the anti-compressive strength of the filling particles and constrained airbag in the right embedded groove.

## 4. Experimental Analysis

The variable-stiffness elastic actuator serves as the core component of a flexible robot, playing a crucial role in determining the overall performance of the flexible robot. One experimental analysis sheds light on the elongation and stiffness of the variable-stiffness elastic actuator, providing the basis for the subsequent development of flexible arm joints. Table 2 details the parameters of the actuator. Specifically, the pressurized and constrained airbags are made from silicon–fluorine rubber tubes, the constrained spiral tube is made from 304 stainless steel, and diamond sand with a 1 mm diameter is used as the filler material.

### 4.1. Anti-Tensile Stiffness Experiment

**Figure 9:**
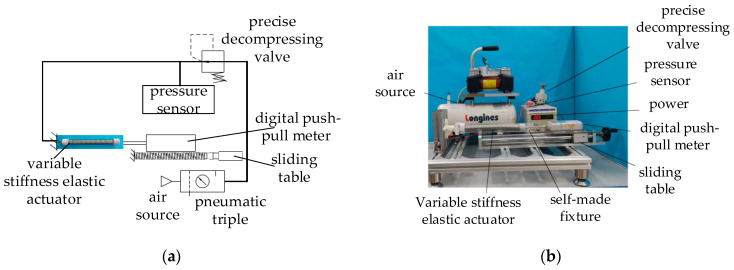
Anti-tensile (anti-compressive) stiffness experimental system: (**a**) experimental principle; (**b**) experimental device.

The experimental principle of the anti-tensile stiffness of the variable-stiffness elastic actuator is shown in Figure 9a, and the corresponding experimental device is shown in Figure 9b. During the experiment, the precise decompressing valve was used to control the air pressure value of the actuator. A laser displacement sensor was used to measure the displacement of the actuator, and a digital push–pull meter was used to obtain the external force at different air pressures. Table 3 gives the parameters of the experimental system components. 

Seven limiting surfaces are set according to the elongation during the experiment. The elongation range is 0–30 mm, the increment is 5 mm, and the corresponding air pressure range is 0–0.21 MPa. The anti-tensile stiffness experiment was carried out for each limit surface. The horizontal movement of the digital push–pull meter was driven by a linear slide. Axial tension was applied to the actuator to generate a fixed axial displacement of 10 mm. The laser displacement sensor was used to measure the displacement of the actuator, and the digital push–pull meter was used to obtain the axial tension. The average value of the five experiments was taken to obtain the following experimental data.

**Figure 10:**
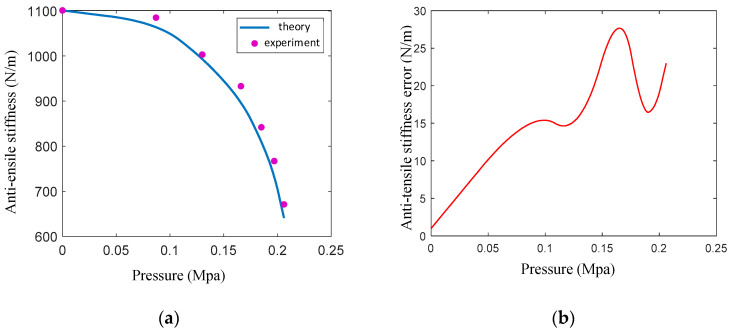
Comparison of theory and experiment and relative error of anti-tensile stiffness: (**a**) comparison of theory curve and experiment data; (**b**) relative error of anti-tensile stiffness.

After introducing the driving force correction coefficient K_A_ = 1.43, the theoretical curve of the anti-tensile stiffness of the variable-stiffness elastic actuator is consistent with the change trend of the experimental data (Figure 10). The maximum relative error is 3.4%, indicating that the theoretical model can reflect the variation in the anti-tensile stiffness with the air pressure. The anti-tensile stiffness of the actuator decreases nonlinearly with the increase in air pressure, which is mainly affected by structural and material properties. However, under the same air pressure, the stiffness of the actuator is greatly improved compared with the artificial-muscle actuator without variable-stiffness structure. The anti-tensile stiffness at the initial position is 1101 N/m, and the anti-tensile stiffness at 0.21 MPa is 641 N/m. When the air pressure exceeds 0.21 MPa, the actuator reaches the maximum elongation, and the anti-tensile stiffness of the actuator depends on the anti-tensile strength of the spiral tube.

### 4.2. Anti-Compressive Stiffness Experiment

The anti-compressive stiffness experiment is carried out using the experimental principle and experimental device shown in Figure 9. When the pressure is 0.17 MPa, the filling particles are completely embedded in the groove of the spiral tube. The anti-compressive stiffness of the actuator depends on the anti-compressive strength of the filling particles. Therefore, the pressure range is 0–0.17 MPa during the experiment. Five limiting surfaces are set according to the elongation, and the elongation range is 0–18 mm. The average value of the five experiments is taken in order to obtain the following experimental data.

**Figure 11:**
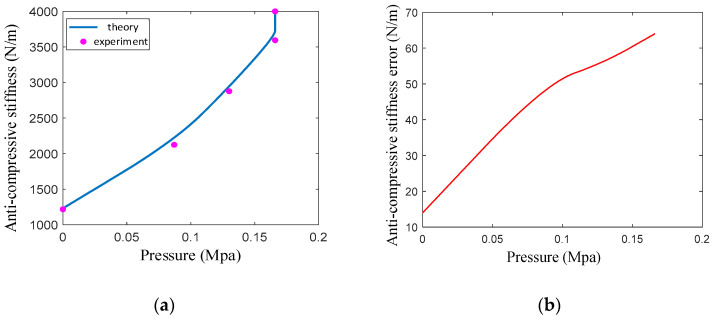
Comparison of theory and experiment and relative error of anti-compressive stiffness: (**a**) comparison of theory curve and experiment data; (**b**) relative error of anti-compressive stiffness.

After introducing the correction coefficient, the theoretical curve of the anti-compressive stiffness is consistent with the trend of the experimental data (Figure 11), and the maximum relative error is 1.8%. The anti-compressive stiffness of the actuator increases nonlinearly with the increase in air pressure; because elongation increases, the embedded depth of the particles becomes larger, and the resistance moment of the wedge increases. The anti-compressive stiffness at the initial position is 1230 N/m, and the anti-compressive stiffness at 0.17 MPa is 3694 N/m, which is three times that of the initial position. When the air pressure exceeds 0.17 MPa, the stiffness increases steeply, driven by the anti-compressive strength of the fully embedded filler particles within the spiral groove.

### 4.3. Lateral-Stiffness Experiment

**Figure 12:**
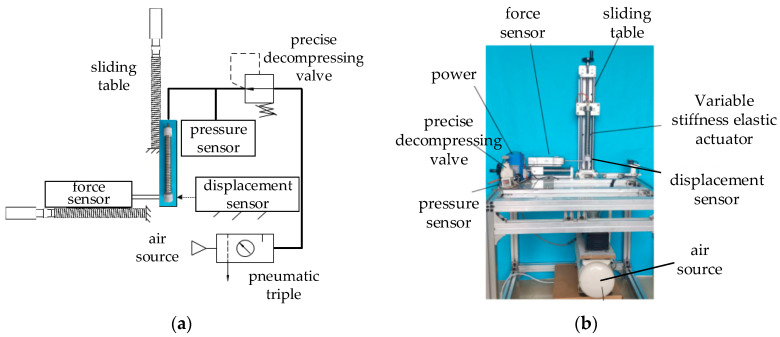
Lateral-stiffness experimental system of the variable-stiffness elastic actuator: (**a**) experimental principle; (**b**) experimental device.

We conducted a lateral-stiffness experiment on the variable-stiffness elastic actuator, as detailed in Figure 12. To mitigate gravitational effects, the actuator was oriented vertically, while a digital push–pull meter was placed horizontally. The actuator’s air pressure was controlled within a range of 0 to 0.17 MPa, corresponding to an elongation range of 0 to 18 mm. For each of the five defined limiting surfaces, we performed a lateral-stiffness test. Axial tension was applied to the actuator to generate a fixed axial displacement of 10 mm. The average value of the five experiments is taken to obtain the following experimental data.

**Figure 13:**
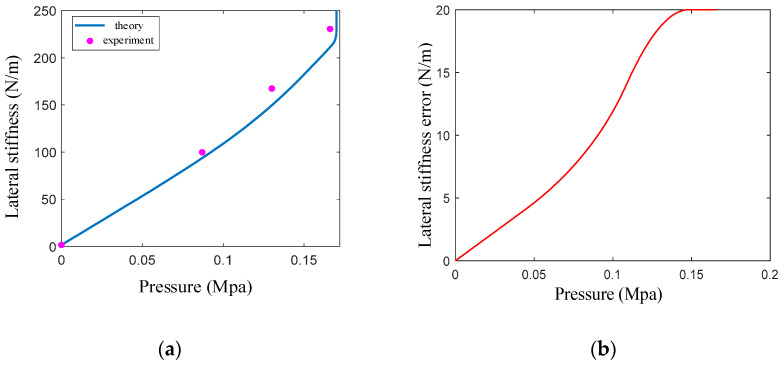
Comparison of theory and experiment and relative error of lateral stiffness: (**a**) comparison of theory curve and experiment data; (**b**) relative error of lateral stiffness.

After introducing the driving force correction coefficient, the theoretical curve for lateral stiffness closely matched the experimental data (Figure 13), and the maximum relative error was 8.7%. Under air pressures below 0.17 MPa, the lateral stiffness increased nonlinearly due to a corresponding increase in elongation, embedded particle depth, and wedge resistance moment. Starting from an initial stiffness of 1.65 N/m, the lateral stiffness surged to 201.3 N/m at an air pressure of 0.17 MPa—an increase of 121 times. When the pressure exceeds 0.17 MPa, the stiffness increases steeply, driven by the anti-compressive strength of the fully embedded filler particles within the spiral groove.

### 4.4. Dynamic Response Experiment

The dynamic experiment of the actuator was carried out to analyze the response of the actuator under step signal excitation. During the experiment, one end of the actuator was fixed and installed on the test bench, and the other end was freely deformed under the excitation of the air pressure signal. The 3D capture system was placed vertically at 3 m in front of the actuator, and the pose remained unchanged. The marker points of the 3D capture system device were uniformly attached to the actuator. The air pressure control system applied a step signal excitation to the actuator. 

**Figure 14:**
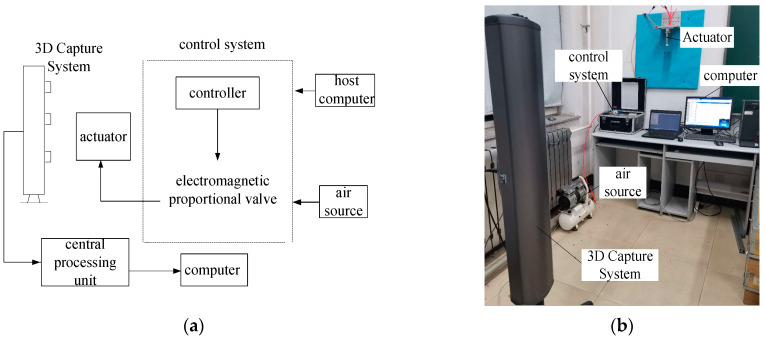
Dynamic experimental system of the variable-stiffness elastic actuator: (**a**) experimental principle; (**b**) experimental device.

The host computer outputs the step signal to the electromagnetic proportional valve to control the pressure of the actuator to elongate. The 3D capture system captured and extracted the dynamic data of the marker points and analyzed the dynamic response of the signal to the motion of the actuator. Figure 14 shows the experimental principle and experimental device. Table 4 gives the parameters of the control system components.

**Figure 15:**
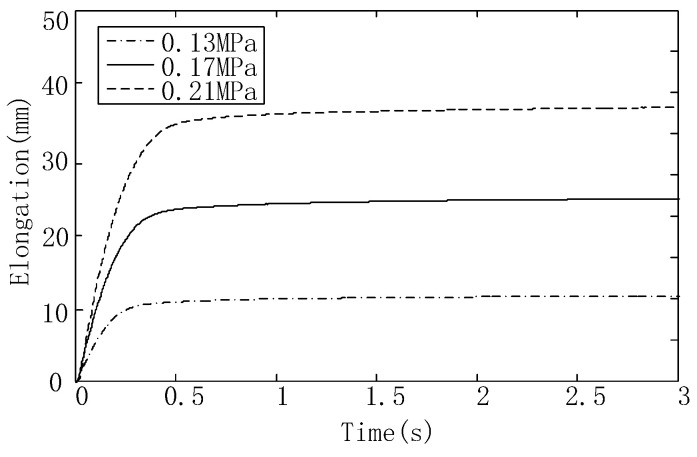
Step response curve of the variable-stiffness elastic actuator.

The control module and the electromagnetic directional valve were used to apply pneumatic excitation to the actuator, and the fuzzy PID control algorithm was used to carry out the response experiment. the elongations of the actuator were recorded at 0.13 MPa, 0.17 MPa, and 0.21 MPa, and the response curve of the actuator was drawn, as shown in Figure 15. 

It can be seen from Figure 15 that with the increase in air pressure, the elongation of the actuator and the response rate increase. At 0.13 MPa, the time required to reach a steady state is 0.24 s; at 0.17 MPa, the time required to a reach steady state is 0.36 s; and at 0.21 MPa, the time required to reach steady state is 0.48 s.

## 5. Conclusions

In summary, by combining the working principles of particle blocking, wedge structures, and pneumatic driving, we developed a variable-stiffness elastic actuator and established theoretical models for anti-tensile stiffness, anti-compressive stiffness, and lateral stiffness and verified them using related experimental tests. The relative error between the theoretical curve and the experimental data is small, which verifies the correctness of the theoretical model.

(1) The anti-tensile stiffness of the actuator decreases with the increase in the air pressure when the air pressure is less than 0.21 MPa and the anti-tensile stiffness is very large, depending on the anti-tensile strength of the spiral tube when the air pressure is greater than 0.21 MPa.
(2) The anti-compressive stiffness increases with the increase in air pressure. When the air pressure is 0.17 MPa, the stiffness is three times that when unpressurized, and the anti-compressive stiffness depends on the anti-compressive strength of the filling particles when the air pressure exceeds 0.17 MPa.
(3) The lateral stiffness increases nonlinearly and is positively correlated with air pressure. When the air pressure is 0.17 MPa, the stiffness is 121 times higher than that when unpressurized, and this stepwise increase in stiffness continued beyond this pressure point.
(4) The variable-stiffness elastic actuator can be applied to flexible robots, which can be used as a driving device and also as a variable-stiffness device to realize the integration of the structure and driving device, while improving the stiffness of the robot.

## Figures and Tables

**Table 1:** Size and material parameters of the variable-stiffness elastic actuator.

Parameter Name	Value		Material
Overall length	230 mm		
Effective length of airbag	180 mm		
Diameter of pressurized airbag	Inner diameter: 8 mm		Silicon–fluorine rubber
	Outer diameter: 12 mm		
Diameter of constrained airbag	Inner diameter: 20 mm		Silicon–fluorine rubber
	Outer diameter: 24 mm		
Diameter of constrained spiral tube	Inner diameter: 25 mm		304 stainless steel
	Outer diameter: 29 mm		
	Maximum length: 216 mm		
	Minimum length: 180 mm		
	Maximum groove size	Length: 3.8 mm	
		Height: 4 mm	
Filling particles	Diameter: 1 mm		Diamond sand
	Number: 36,170		

**Table 2:** Parameters of the variable-stiffness elastic actuator.

Parameter Name	Value
Elastic modulus of airbag *E*	1.042 MPa
Friction coefficient $f_{1}$	0.12
Friction coefficient $f_{2}$	0.13
Friction coefficient $f_{2}$	0.1
Friction coefficient $f_{2}$	0.13

**Table 3:** Experimental system component parameters.

Component Name	Model	Precision
Pump	WX1.5HP	0–0.9 MPa
Precise decompressing valve	IR2020-02	0.001 MPa
Laser displacement sensor	HG-C1030	0.01 mm
Digital push–pull meter	HF-100	0.001 N

**Table 4:** Control system component parameters.

Component Name	Model	Value
Electromagnetic proportional valve	SMC-ITV0030-2MN	0–1.0 MPa
Air pressure sensor	SMC-ISG40A-W1-R-M	0.001 MPa
Three-dimensional capture system	NDI Optotrak Certus	0.1 mmRMS

## Data Availability

The data presented in this study are available on request from the corresponding author.
